# Identifying an appropriate PCV for use in Senegal, recent insights concerning *Streptococcus pneumoniae*NP carriage and IPD in Dakar

**DOI:** 10.1186/s12879-014-0627-8

**Published:** 2014-12-04

**Authors:** Fatim Ba, Abdoulaye Seck, Mamadou Bâ, Aliou Thiongane, Moussa Fafa Cissé, Khady Seck, Madeleine Ndour, Pascal Boisier, Benoit Garin

**Affiliations:** Medical Laboratory, Pasteur Institute, Dakar, Senegal; Pediatric Unit, Albert Royer Hospital, Dakar, Senegal; Medina Health Center, Dakar, Senegal; Saint Martin Health Center, Dakar, Senegal; Epidemiology Units, Pasteur Institute, Yaounde, Cameroon; Bacteriology Laboratory, Pasteur Institute, Antananarivo, Madagascar

**Keywords:** Streptococcus pneumoniae, Nasopharyngeal carriage, Invasive pneumococcal disease, Serotypes, Antibiotic resistance, Children, Sub-Saharan Africa, Senegal

## Abstract

**Background:**

Since 2000, the Global Alliance for Vaccines and Immunization (GAVI) and WHO have supported the introduction of the Pneumococcal Conjugate Vaccine (PCV) in the immunization programs of developing countries. The highest pneumococcal nasopharyngeal carriage rates have been reported (40-60%) in these countries, and the highest incidence and case fatality rates of pneumococcal infections have been demonstrated in Africa.

**Methods:**

Studies concerning nasopharyngeal pneumococcal carriage and pneumococcal infection in children less than 5 years old were conducted in Dakar from 2007 to 2008. Serotype, antibiotic susceptibility and minimum inhibitory concentrations were determined. In addition, among 17 overall publications, 6 manuscripts of the Senegalese literature published from 1972 to 2013 were selected for data comparisons.

**Results:**

Among the 264 children observed, 132 (50%) children generated a nasopharyngeal (NP) positive culture with *Streptococcus pneumoniae*. The five most prevalent serotypes, were 6B (9%), 19 F (9%), 23 F (7.6%), 14 (7.6%) and 6A (6.8%). Fifteen percent of the strains (20/132) showed reduced susceptibility to penicillin and 3% (4/132) showed reduced susceptibility to anti-pneumococcal fluoroquinolones. Among the 196 suspected pneumococcal infections, 62 (31.6%) *Streptococcus pneumoniae* were isolated. Serogroup 1 was the most prevalent serotype (21.3%), followed by 6B (14.9%), 23 F (14.9%) and 5 (8.5%). Vaccine coverage for PCV-7, PCV-10 and PCV-13, were 36.2% (17/47), 66% (31/47) and 70.2% (33/47) respectively. Reduced susceptibility to penicillin and anti-pneumococcal fluoroquinolones was 6.4% and 4.3%, respectively, and the overall lethality was 42.4% (14/33).

**Conclusions:**

This study confirms a high rate of carriage and disease caused by *Streptococcus pneumoniae* serotypes contained within the current generation of pneumococcal conjugate vaccines and consistent with reports from other countries in sub-Saharan Africa prior to PCV introduction. Antimicrobial resistance in this small unselected sample confirms a low rate of antibiotic resistance. Case-fatality is high. Introduction of a high valency pneumococcal vaccine should be a priority for health planners with the establishment of an effective surveillance system to monitor post vaccine changes.

**Electronic supplementary material:**

The online version of this article (doi:10.1186/s12879-014-0627-8) contains supplementary material, which is available to authorized users.

## Background

In developed nations, a substantial decrease in the invasive pneumococcal disease (IPD) incidence rate has been achieved through the introduction of the 7-valent- pneumococcal conjugated vaccine (PCV-7) into pediatric immunization programs. PCV-10 and PCV-13 including additional pneumococcus serotypes are now gradually replacing PCV-7 in immunization programs mainly in developed countries [1],[2] but also in developing countries [3],[4]. In contrast, the incidence of IPD has changed little in developing countries (DC), reflecting poor access to pneumococcal vaccination and the fact that PCV-7 does not contain serotypes 1, 5 and 6A known to be prevalent in Africa [5]. Nevertheless, the funding received from the Global Alliance for Vaccines and Immunization (GAVI) since 2000 and WHO recommendations for PCV use since 2007 [6] have increased the introduction of PCV in national immunization programs. In December 2012, the PCV introduction among WHO member states reached 41% (19/46) in Africa [6], and both Gambia [7],[8] and Kenya [3],[4] are currently evaluating the national impact of PCV initiatives on carriage prevalence and serotype distribution.

Most *Streptococcus pneumoniae* (*S. pneumoniae*) serotypes colonize the nasopharyngeal niche as commensal inhabitants and subsequently migrate to cause IPD [9]. The most commonly observed serotypes in nasopharyngeal (NP) carriage worldwide, 6B, 19 F, and 23 F, are less invasive, but are also more likely to harbor antibiotic resistance [5], whereas serotypes 5, 7 F, and 1 are infrequent colonizers that are more invasive, especially in Africa [10]. Young children in DC, where overcrowding is typical, are risk groups for this dissemination, showing the highest carriage rates worldwide (40-60%) [5],[11].

Since 2000, the pneumococcal infection burden in Africa has been a source of great concern, as this continent has the highest incidence of pneumococcal infections (3627 per 100000) and pneumococcal mortality (399 per 100000), with case fatality rates of 11%, 73% and 58% for pneumonia, meningitis and non-pneumonia non-meningitis diseases, respectively [12].

In West African countries, serotype 1 is the most prevalent serotype in IPD, followed by serogroups 5, 6, 2, 3 and 12 [13]; however, this serogroup distribution might be different according to the country, therefore the serotype coverage in this region varies. For example, the serotype coverage ranges from 39% to 80% for PCV10 [14]. In countries where pneumococcal vaccination has been initiated, the surveillance of pneumococcal isolates remains a necessity with regard to pneumococcal carriage and disease, antibiotic resistance prevalence rate, capsular switching and vaccine escape [10].

The prevalence of Penicillin Non-Susceptible Pneumococcus (PNSP) has increased worldwide, associated with the spread of specific clones with serotypes 6B, 9 V, 14, 19 F, and 23 F. After the introduction of PCV-7 a reduction in antibiotic-resistant infections has been observed, reflecting a decline in the incidence of IPD caused by PCV-7 serotypes. To go beyond the disease protection capacity of PCV-7, extended valency vaccines have been developed which include 3 and 6 additional capsular polysaccharides (1, 3, 5, 6A, 7 F and 19A). These PCVs designated PCV-10 and PCV-13 are gradually used and licensed in many countries around the world. These implementations provide the opportunity to more deeply investigate the impact these vaccines will have on disease [1],[15].

In the present study, we performed a PubMed search for studies on *S. pneumoniae* infection and NP carriage in Senegal published from January 1, 1972 to September 30, 2013 and selected pediatric studies for comparison with our current findings. Herein, we report the serotype distribution and antibiotic resistance of *S. pneumoniae* isolated from NP carriage and IPD patients, and these data were compared with previous data from studies conducted in Senegal. Moreover, the question of the most appropriate PCV for Senegal is discussed regarding the *S.pneumoniae* serotype distribution in carriage and IPD.

## Methods

### Ethics statement

This study was approved through the Ethics Committee of the Ministry of Health. Written consent was obtained from parents or legal guardians before inclusion. A detailed description of the study was provided in the native language for illiterate individuals and for both studies different questionnaires were conducted.

### Population study

This study was conducted in Dakar, the capital of Senegal, in 2007 and 2008. All samples were collected from children less than five years of age. Experiments involving pneumococcal carriage were conducted at 2 Health Centers located in Medina and Saint Martin in Dakar between March 2007 and October 2008. These two Health Centers have been chosen for their localization in popular areas and their capacities to draw a large population of children from surrounding areas. Medical activities comprise primary care, vaccinations, medical consultations and hospitalizations.

Children consulting for vaccination were asked to provide a nasopharyngeal swab sample (Calgiswab, VWR). During the 20 month period of the study, in both Health Centers, children were sampled on nine different months over the study period, two days per month, on a total of 18 days. Children were sampled once and very few refusals were recorded. Exclusion criteria were presence of antibiotic consumption within the preceding 2 weeks.

In the clinical study, during 21 months, from January 2007 to September 2008, children with suspected *S. pneumoniae* disease were recruited in the pediatric wards from the Albert Royer/Fann University Hospital. The Albert Royer hospital is the referral pediatric center in Dakar and children attending these pediatric units originate from the city of Dakar and its outskirts, but also from more remote areas outside the capital.

An IPD case was defined as culture of *S. pneumoniae* from a normally sterile body site as blood, CSF or pleural effusion. The diagnosis of meningitis was based on *S. pneumoniae* identified in CSF through culture. Septicemia was defined as *S. pneumoniae* cultured in blood with no distinctive clinical syndrome. The diagnosis of empyema was based on cultures positive for *S. pneumoniae* that were taken from pleural fluids. Repeat samples from sterile sites from the same child were regarded as part of the same episode and one isolate was included if both isolates expressed the same serotype. Specimens were sampled in accordance with the clinical pattern; here are reported results of blood-cultures, CSF, and pleural fluids.

### Laboratory experiments

The Albert Royer hospital medical laboratory processed the bacterial cultures of all the specimens sampled from hospitalized children. *S. pneumoniae* isolates were then sent to the Pasteur Institute laboratory for susceptibility testing and serotyping. *S. pneumoniae* was isolated and identified using common bacteriological methods. The suspected samples were streaked onto fresh sheep blood agar plates (5%) containing optochin disks and incubated at 35-37°C with 5% CO_2_ for 24 h; in cases of negativity, the cultures were incubated up to 48 h. Bacteria showing inhibition zones around the optochin disks were subjected to additional tests, such as gram staining, catalase reaction and latex agglutination (Slidex Pneumo-Kit®, bioMérieux). The capsular type was determined for 23 different serogroups through latex agglutination (Pneumotest kit, Statens Serum Institute, Copenhagen Denmark). Then when needed, traditional factor antisera were used to serotyping the PCV-7 serogroups 6, 9, 18, 19 and 23 by the Quellung reaction. The isolates showing no reactivity were defined as “Non Typed” (NT) strains. Beta-lactam (BL) and Fluoroquinolone (FQ) susceptibility was examined using the disk-diffusion method according to the Comité de l’Antibiogramme de la Société Française de Microbiologie (CASFM 2008, http://www.sfm-microbiologie.org). CASFM and EUCAST (European Committee on Antimicrobial Susceptibility Testing, http://eucast.org) guidelines recommend the use of different oxacillin disk screening tests for detection of penicillin resistant pneumococci, a 5-μg oxacillin disk for CASFM and a 1-μg oxacillin disk for EUCAST. As a result, cut-off values of the inhibition zone are 26 mm and 20 mm, respectively. In cases of intermediate or resistant susceptibility, the Minimum Inhibitory Concentration (MIC) was determined for BL and FQ using a standard agar diffusion method (Steer’s multipoint apparatus). The Steer’s device is an inoculum replicator with multipoints delivering simultaneously up to 96 dots of the prepared bacterial suspension on the surface of the agar plates [16].

### Senegalese literature

From January 1, 1972 to September 30, 2013, 17 publications on pneumococcal infections in Dakar were retrieved from PubMed using *S. pneumoniae*, infection, nasopharyngeal carriage, Dakar, Senegal, and Africa as keywords. Six of the 17 publications including available results concerning children less than 5 years of age, 4 in French and 2 in English, were selected for further analysis.

### Statistical analysis

Proportions were calculated with 95% Confidence Interval [CI] when relevant. Mean age values and sex ratios were estimated on children from both the carriage and the invasive disease studies.

## Results

### Nasopharyngeal carriage study

**Figure 1:**
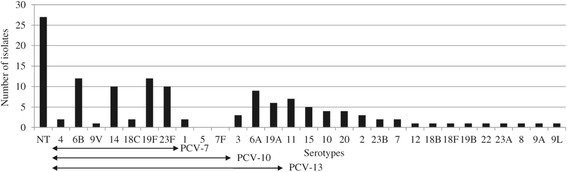
**Serotype distribution of S. pneumoniae isolates from NP carriage in children ≤24 months of age attending 2 health care centers in Dakar, Senegal.** The serotypes are listed on the X-axis, starting with the NT, and followed by the serotypes covered by PCV-7, PCV-10 and PCV-13. The NVT are listed consecutively from serotype 11 to serotype 8 by decreasing order.

**Figure 2:**
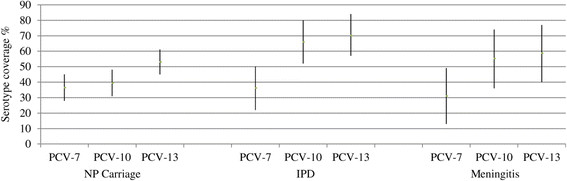
**PCV-7, PCV-10 and PCV-13 vaccine coverage in** ***S. pneumoniae*** **NP carriage, IPD and meningitis.** PCVs are listed on X-axis by NP carriage, IPD and Meningitis. 95% Confidence Intervals are shown for each PCV vaccine coverage.

The Nasopharyngeal (NP) carriage study included 264 children less than 24 month of age; 253 (95.8%) children were between 0 and 12 month of age, and the mean age was 5 months, with a sex ratio (M/F) of 1.13. Among the 264 children, 132 (50%; 95% CI, 44-56%) children had NP positive *S. pneumoniae* cultures. *S. pneumoniae* NP positive carriers were observed as early as the first month after delivery. The serotype distribution displayed 28 different serotypes. The five most prevalent serotypes were 19 F (9%, 12/132), 6B (9%, 12/132), 14 (7.6%, 10/132), 23 F (7.6%, 10/132), and 6A (6.8%, 9/132), accounting for 40.2% of the overall serotypes (Figure 1). Serotype 1 was detected in 2 of the 132 children. PCV-7 covered 48/132 (36.4%), PCV-10 covered 52/132 (39.4%) and PCV13 covered 70/132 (53%) of the identified serotypes (Figure 2).

According to CASFM, among the 132 isolates, 70 showed an inhibition diameter of less than 26 mm on the oxacillin disk (5 μg), 20 (15%; 95% CI, 9-22%) of the isolates showed reduced susceptibility to penicillin by MIC determination (MIC ≥ 0.12 mg/L) and 2 of the 20 isolates were resistant to penicillin (>2 mg/L). The first strain, of NT serotype (penicillin MIC = 4 mg/L), was also amoxicillin resistant (MIC = 4 mg/L) and cefotaxime non-susceptible (MIC = 1 mg/L), and the second strain, of 23 F serotype (penicillin MIC = 8 mg/L), was amoxicillin and cefotaxime susceptible. The mean value of penicillin MIC among the 20 PNSP was 0.33 mg/L. Regarding FQ resistance among the 132 isolates, 32 *S. pneumoniae* isolates showed a reduced susceptibility to ciprofloxacin, of which 10 isolates (7.6%, 10/132) were completely resistant to this antibiotic (MICs 4 to 8 mg/L), while 3 isolates were levofloxacin resistant (MIC = 4, 4 and 64 mg/L) and 1 isolate was moxifloxacin resistant (MIC = 1 mg/L).

### Invasive pneumococcal disease study

During the 21 month IPD study, 196 children between 0 and 5 years of age were included; the mean age was 15.5 months, with 54.6% (107/196) of the children less than 1 year old, and the sex ratio (M/F) was 1.3.Two hundred and forty specimens were collected, 144 bloodstreams, 75 CSF and 21 pleural fluid (PF) samples. Among 101 children with blood samples, 74 children with CSF samples and 21 children with PF samples, 12 septicemia cases (12/101, 11.9%), 42 meningitis cases (42/74, 56.8%) and 8 empyema cases (8/21, 38%) were observed. Sixty two *S. pneumoniae* infected children out of the 196 suspected children were finally included (31.6%).

**Figure 3:**
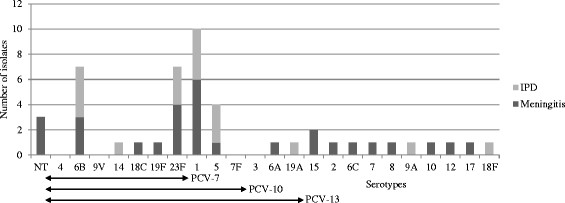
**Serotype distribution by IPD and meningitis in children ≤5 years of age attending pediatric units in Albert Royer hospital in Dakar, Senegal.** The serotypes are listed on the X-axis, starting with the NT, and followed by the serotypes covered by PCV-7, PCV-10 and PCV-13. The NVT are listed consecutively from serotype 15 to serotype 18 F.

Antibiotic susceptibility testing and isolate serotyping were performed on 47 (75.8%) of the 62 *S. pneumoniae* isolates: 29 meningitis cases, 10 septicemia cases and 8 empyema cases. Twenty different serotypes were identified (Figure 3). Serotype 1 was the most prevalent serotype (10/47, 21.3%; 95% CI, 9-33%), followed by 6B (14.9%, 7/47) and 23 F (14.9%, 7/47) and 5 (8.5%, 4/47).

Among the 29 *S. pneumoniae* meningitis, 16 different serotypes were identified, and serotypes 1 (6/29, 20.7%; 95% CI, 5-36%), was the most prevalent followed by 23 F (4/29, 13.8%) and 6B (3/29, 10.3%) (Figure 3).

The mean vaccine coverage was 36.2% (17/47) with PCV-7, 66% (31/47) with PCV-10 and 70.2% (33/47) with PCV-13 (Figure 2). The meningitis vaccine coverage was 31% (9/29) with PCV-7, 55.2% (16/29) with PCV-10 and 58.6% (17/29) with PCV-13 (Figure 2).

Three isolates showed reduced susceptibility to penicillin, and these bacteria were isolated from meningitis cases. Among the 3 PNSPs, one isolate was resistant to penicillin (MIC = 16 mg/L), amoxicillin (MIC = 32 mg/L) and cefotaxime (MIC = 32 mg/L). Among the 17 isolates with reduced susceptibility to ciprofloxacin (36.2%; MIC > 0.12 mg/L), 2 isolates were moxifloxacin (MIC = 4 and 16 mg/L) and levofloxacin resistant (MIC = 32 mg/L). The isolate simultaneously resistant to BL and FQ was of serotype 1, and the second FQ resistant isolate was of serotype 5.

The outcomes were known for 33 children out of the 62 children (53.2%) and 22 children out of the 42 meningitis cases with *S. pneumoniae* positive results, showing an overall lethality of 42.4% (95% CI, 25-60%) (14/33) and a meningitis lethality of 54.5% (95% CI, 32-76%) (12/22).

Regarding the 6 Senegalese publications, one study concerned both NP carriage and Respiratory Tract Infections (RTI) [17], one study focused on NP carriage [18] and four studies concerned meningitis and invasive infections [19]-[22]. The serotype results were available in one NP carriage study, and susceptibility testing and lethality were assessed in 3 studies.

## Discussion

### Carriage

The 50% (132/264) mean NP carriage prevalence rate observed in the present study is consistent with the results obtained in most of the publications concerning young children [5],[15]; indeed, Echave et al. in 2003 [17] reported a prevalence rate of 56% in Senegal. However, Baylet et al. reported higher NP carriage prevalence rates in rural (77%) and urban (69.6%) areas [18] in Senegal and other African countries. In Kenya, the NP carriage was 65.8% in children less than 5 years old and 79% in children 6–11 months of age [23]. In Nigeria, infants less than 2 years showed NP prevalence rates of 74.4%, with a peak value between 6–9 months [24], and in Burkina Faso [25], the NP carriage rate was 69.4% in children less than 1 year of age versus the 48% carriage rate observed in the present study.

The high NT prevalence rate observed in our study is partially due to the Pneumotest-kit used for serotype determination in the laboratory, with only 23 different serogroups identified. This corresponds to non-typed strains rather than to non-typable strains. The most prevalent carriage serotypes found in this study were 6B, 19 F, 23 F, 14, 6A, 11 and 19A (Figure 1), and some (6B, 23 F) of these serotypes were concurrently observed in IPD (Figure 3). This serotype distribution (19 F, 6B, 6A, 23 F), is similar to that reported in Ghana [26], Kenya [23] and Nigeria [24].

The determination of serotype coverage showed, a similar Senegalese PCV-13 coverage (53%, 95% CI, 45-61%, 70/132) to other African countries, such as Ghana (50%) [26], Kenya (59%) [23], and Nigeria (61.7%) [24].

In Senegal, the 15% PNSP prevalence rate observed in the present study, with a high proportion of intermediate resistance (90%), is consistent with the results of the Echave et al. study conducted in a Senegalese rural area in 2003 (13%) [17]. However, at that time, MIC testing on PNSP was not performed, and the 13% prevalence rate could actually be lower than reported. In Nigeria and in Ghana, rare penicillin-resistance isolates were detected but rather an intermediate resistance to this antibiotic was observed, 30.9% and 45% respectively [24],[26]. In Senegal, the prevalence of anti-pneumococcal FQ resistance (moxifloxacin and levofloxacin) was 3% (4/132), with a serotype 11 isolate showing a high levofloxacin MIC (64 mg/L). These results suggest that *S. pneumoniae* antibiotic resistance in NP carriage is not a major concern in Senegal.

### IPD

Our study has limitations due to single hospital site recruitment; however we believe that our surveillance captures most laboratory-confirmed IPD cases and, because pediatric wards in the Albert Royer hospital welcome a large population of children living in the Dakar region, enables comparison with other published data from Senegal or other African countries.

The study is also limited by the incomplete serotyping and susceptibility testing of overall cultured isolates. Given these limitations, results have to be discussed cautiously, but in case more isolates had been tested, we are unlikely to see contradictory findings as these data are consistent with previous Senegalese and sub-Saharan Africa published results.

The most prevalent serotypes were serotype 1 (21.3%), 6B (14.9%), 23 F (14.9%) and 5 (8.5%) in accordance with what is known in Africa [14] (Figure 3). Dia et al. reviewed 103 isolates from invasive and non-invasive diseases collected in Senegal during 2 distinct periods (1996–1999 and 2007–2010) and reported findings similar to those of the present study, with 1, 23 F, 5 and 6B as the most frequent serotypes in IPD [22]. In West African countries, the major serotypes in IPD in children less than 5 years of age are 5, 6A, 1 and 14, and in Burkina Faso and Senegal the three most prevalent serotypes are 1, 5 and 6 [14].

The vaccine coverage observed in the present study (Figure 2) is consistent with the results of Donkor et al., reporting West African serotype coverage of 2%–36% for PCV7, 39%–80% for PCV10, and 65%–87% for PCV13 [14].

**Figure 4:**
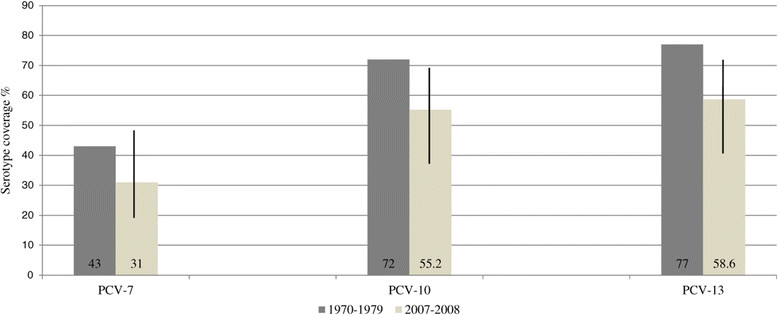
**Meningitis serotype coverage comparisons between 1970–1979 and 2007–2008.** PCVs are listed on the X-axis. 95% Confidence Intervals for serotype coverage are shown for the 2007–2008 period.

Among the 29 *S. pneumoniae* meningitis tested with 28 different antisera, 16 different serotypes were identified, and serotypes 1 (6/29, 20.7%; 95% CI, 5-36%), was the most prevalent followed by 23 F (4/29, 13.8%), and 6B (3/29, 10.3%) (Figure 3). Serotype data have been obtained in 3 previous studies on meningitis in Senegal [19]-[21]. In the 70s the most frequent serotypes were 5, 6, 23 and 12, although serotype 1 was also detected (4-8%). Dia et al. reported similar results to the present study, with serotype 1 as the leading serotype in meningitis between 2006 and 2010, followed by 23 F, 5, and 6B. Differences between the serotype distribution reported in the 70s and the current findings involve serotypes 1 and 5, as the prevalence of these bacteria has changed with time. Interestingly, the meningitis serotype coverage has not changed since the 70s [20] (Figure 4); however, at this time the serotypes were only examined at the serogroup level, potentially resulting in an overestimation of the vaccine coverage rate. Meningitis serotype coverage rates for PCV-10 and PCV-13 are 55.2% (95% CI, 36-74%) and 58.6% (95% CI, 40-77%), respectively, and these values are similar to the meningitis vaccine coverage rate reported in the Togo and the Burkina Faso for PCV-10 and 13 (53%) [27].

In our study, 4 PNSPs were isolated from 53 IPD cases, the PNSP prevalence rate is 7.5% (95% CI, 0-15%). In 2003, Echave et al. reported an 18% PNSP prevalence rate in RTI, with no MIC determination to distinguish penicillin intermediate and resistant isolates in Senegal [17]. Dia et al. [22] did not report any information concerning PNSPs. A meta-analysis was conducted in 2013 to examine the antibiotic susceptibility of community acquired invasive infections from 1978 to 2011 in Africa [28]. This publication reports antibiotic resistance rates in IPD of 19.3% to penicillin, 8.6% to amoxicillin, and 0.9% to ceftriaxone and concluded that the majority of pneumococci remains susceptible to amoxicillin. Regarding beta-lactamin antibiotic resistances in Senegal the situation is similar to these findings conversely to Ethiopia where resistance rates were 31.3%, 29% and 9.8% for penicillin, ampicillin and ceftriaxone respectively [29]. Anti-pneumococcal FQ resistance rates (levofloxacin and moxifloxacin) remain moderate for levofloxacin (2/53, 3.8%; 95% CI, 0-9%), but could be higher for moxifloxacin (5/53, 9.4%; 95% CI, 1-18%). Therefore PNSPs are not widespread in Senegal, but serotype 1 isolates with high MIC to BL, associated with FQ resistance are present and emergence of such isolate would be concerning.

Even though outcomes were only known for 53.2% (33/62) of children with *S. pneumoniae* positive results, the high overall case fatality rate (CFR) (44%; 95% CI, 27-61%) and meningitis CFR (54.5%;95% CI, 32-76%) found are consistent with lethality reported in other African countries, with a mean rate of 73% (18%-94%) in meningitis [12]. These CFR are also similar to the results of studies conducted in the 70s in Senegal [20], and this lack of improvement represents a major concern.

Since November 2012, the introduction of PCV-7 has been recommended in Senegal, administered at two doses at 2 and 3 months of life, generating a family cost of 109.2 USD per dose. The invasiveness of individual serotypes is reflected in the case:carrier ratio (CCR) which distinguish the more from the less invasive serotypes [30]. Our study with a low number of serotyped isolates identified, did not fulfill standards allowing CCR assessment. However, PCV-10 additional types (1, 5, 7 F) are considered as having higher CCR than the additional three in PCV-13 (3, 6A, 19A) [31] and our findings could be in agreement with these statements. Indeed, in Dakar, PCV-10 additional types comprise 29.8% (14/47) of IPD isolates but only 1.5% (2/132) of carried isolates and PCV-13 additional valency vaccines comprise 4.2% (2/47) of IPD isolates and 13.6% (18/132) of NP carriage isolates. This observation suggests that in Senegal, PCV-10 or PCV-13 introduction could reduce the incidence of IPD, and additional PCV-13 serotypes (3, 6A, and 19A) might have a greater impact on serotype distribution in carriage than on the reduction of IPD. Regarding the invasiveness of serotypes and the cost effectiveness, PCV-7 could be replaced by PCV-10 instead of PCV-13.

## Conclusions

Although cefotaxime- and FQ-resistant isolates have been detected in Senegal, pneumococcal antibiotic resistance is not a major issue. The primary concern is the CFR, and measures should be urgently implemented in Pediatric wards in Dakar to improve children outcomes. An effective pneumococcal surveillance network should be developed to collect representative data on pneumococcal carriage and infection and to detect serotype changes.

## Authors’ original submitted files for images

Below are the links to the authors’ original submitted files for images. Authors’ original file for figure 1 Authors’ original file for figure 2 Authors’ original file for figure 3 Authors’ original file for figure 4
