# Supplementary figures and images for: Identifying an appropriate PCV for use in Senegal, recent insights concerning Streptococcus pneumoniaeNP carriage and IPD in Dakar
Source: BMC Infect Dis. 2014 Dec 4;14:627. doi: 10.1186/s12879-014-0627-8 (PMC4258793; doi:10.1186/s12879-014-0627-8)

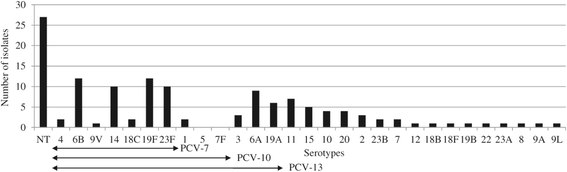

Supplement: Supplementary file 1 — Authors’ original file for figure 1 [file 12879_2014_627_MOESM1_ESM.gif]

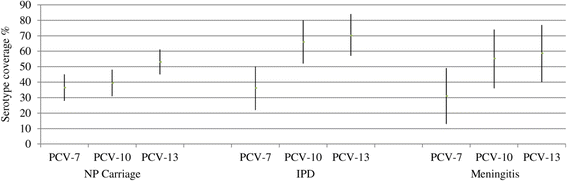

Supplement: Supplementary file 2 — Authors’ original file for figure 2 [file 12879_2014_627_MOESM2_ESM.gif]

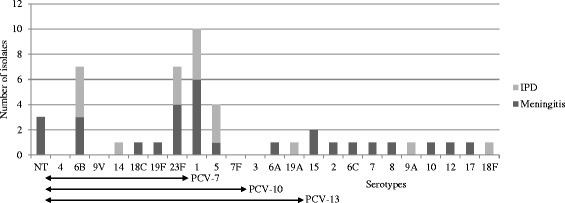

Supplement: Supplementary file 3 — Authors’ original file for figure 3 [file 12879_2014_627_MOESM3_ESM.gif]

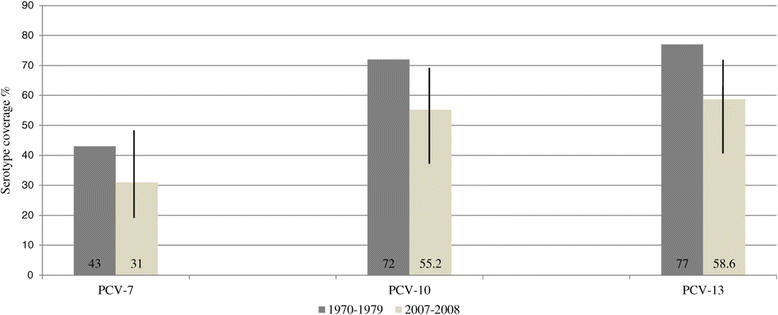

Supplement: Supplementary file 4 — Authors’ original file for figure 4 [file 12879_2014_627_MOESM4_ESM.gif]
